# Complex Refractory Esophageal Stricture Due to Chronic Gasoline Ingestion: A Case Report

**DOI:** 10.3390/medicina59061020

**Published:** 2023-05-24

**Authors:** Henry Sutanto, Amie Vidyani

**Affiliations:** 1 Department of Internal Medicine, Faculty of Medicine, Universitas Airlangga, Surabaya 60132, Indonesia; henry1988md@gmail.com; 2 Department of Internal Medicine, Dr. Soetomo General Academic Hospital, Surabaya 60286, Indonesia; 3 Division of Gastroenterology and Hepatology, Department of Internal Medicine, Faculty of Medicine, Universitas Airlangga, Surabaya 60132, Indonesia

**Keywords:** esophageal stricture, gasoline ingestion, corrosive, gastroenterology, dilation of esophagus, upper gastrointestinal endoscopy, Savary-Gilliard bougie, controlled radial expansion balloon

## Abstract

Esophageal stricture is a narrowing of the esophageal lumen which is often characterized by impaired swallowing or dysphagia. It can be induced by inflammation, fibrosis or neoplasia which damages the mucosa and/or submucosa of the esophagus. Corrosive substance ingestion is one of the major causes of esophageal stricture, particularly in children and young adults. For instance, accidental ingestion or attempted suicide with corrosive household products is not uncommon. Gasoline is a liquid mixture of aliphatic hydrocarbons derived from the fractional distillation of petroleum, which is then combined with additives such as isooctane and aromatic hydrocarbons (e.g., toluene and benzene). Gasoline also contains several other additives including ethanol, methanol and formaldehyde, which make it a corrosive agent. Interestingly, to the best of our knowledge, the incidence of esophageal stricture caused by chronic gasoline ingestion has not been reported. In this paper, we report the case of a patient with dysphagia due to complex esophageal stricture due to chronic gasoline ingestion who underwent a series of esophago-gastro-duodenoscopy (EGD) procedures and repeated esophageal dilation.

## 1. Introduction

Esophageal stricture is a narrowing of the esophageal lumen which is often characterized by impaired swallowing or dysphagia [1,2]. It can be caused by inflammation, fibrosis or neoplasia which causes damage to the mucosa and/or submucosa of the esophagus. The incidence of esophageal stricture has not been widely reported in previous studies. One study reported an incidence of esophageal stricture of 1.1 per 10,000 person-years, which increased with age [1,3]. Peptic stricture is the most common type of esophageal stricture (accounting for 70–80% of cases of esophageal stricture in adults) and is commonly caused by gastroesophageal reflux disease (GERD). Ingestion of corrosive substances is also a major cause of esophageal stricture, especially in children and young adults. Accidental ingestion or attempted suicide by ingestion of corrosive household products is not uncommon. According to data from the American Association of Poison Control Centers (AAPCC), exposure to corrosive substances is one of the five most common causes of poisoning in adults and children under the age of five [4], and the resulting damage can range from mild injury to extensive esophageal necrosis [5]. In addition to these two main causes, esophageal stricture can also be induced by eosinophilic esophagitis, drug-induced esophagitis (e.g., due to non-steroidal anti-inflammatory drugs/NSAIDs, potassium chloride [KCl] tablets and tetracycline antibiotics), radiation injuries, iatrogenic strictures, anastomotic strictures, chemotherapy, temperature injuries and even infections. In addition, esophageal strictures can also be due to malignancy in the esophagus, such as esophageal adenocarcinoma, squamous cell carcinoma or lung malignancy metastases [1].

Corrosive substances are defined as materials that can attack and destroy living tissue, organic compounds and metals through chemical reactions. The more acidic or alkaline a substance is, the more effective it is as a corrosive agent. Some examples of corrosive substances are hydrochloric acid (HCl), sulfuric acid (H_2_SO_4_), nitric acid, chromic acid, acetic acid, ammonium hydroxide (NH_4_OH), potassium hydroxide (KOH), sodium hydroxide (NaOH) and sodium hypochlorite (NaClO). Gasoline is a liquid mixture of aliphatic hydrocarbons derived from fractional distillation of petroleum, which is combined with additives such as isooctane and aromatic hydrocarbons (e.g., toluene and benzene). Gasoline also contains several other additives including ethanol, methanol, formaldehyde, xylene, 1,3-butadiene, methyl tert-butyl ether (MTBE), and hexane [6]. As previously reported, acute ingestion of toluene can cause irritation, corrosion and injury to the gastrointestinal tract, which manifests as abdominal pain, nausea, vomiting and vomiting of blood [7]. In addition, the ethanol content in gasoline may increase the ability of gasoline to absorb water and will make gasoline corrosive [8]. Interestingly, the incidence of esophageal stricture caused by chronic gasoline ingestion has not been reported. In this paper, we report the case of a patient with dysphagia due to complex esophageal stricture due to chronic gasoline ingestion who underwent a series of esophago-gastro-duodenoscopy (EGD) procedures and repeated esophageal dilation.

## 2. The Case Description

A 50-year-old woman came to the emergency room (ER) at the Dr. Soetomo General Academic Hospital in Surabaya, Indonesia with a chief complaint of vomiting every time she ate and drank. Vomiting was experienced by the patient a few seconds after eating or drinking without being preceded by nausea. The vomit contained food or drink consumed seconds before. This complaint was experienced by the patient 3 weeks before coming to the ER. The patient also had difficulty swallowing which had gotten worse 5 months before the ER visit. When she was in the emergency room, the patient complained of not being able to consume any liquids, including water. The patient admitted that she had lost more than 20 kg in the last 5 months. The patient defecated approximately once every 2–3 days. The patient’s urination was normal. Any history of fever, cough, runny nose, pain when swallowing, tightness and the appearance of a lump in the body, especially in the neck, was denied. History of a burning sensation in the chest that rose to the throat and gastric acid reflux, especially when lying down or sleeping at night, was denied. There was a history of hypertension, and the patient claimed not to have diabetes mellitus, heart disease or other chronic diseases.

**Figure 1:**
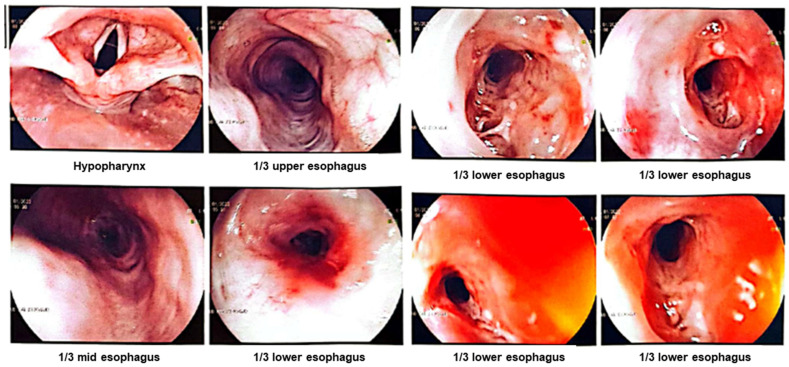
The narrowed and easily bled esophageal lumen was documented during the EGD at Sidoarjo General Hospital.

The patient had previously been examined at Sidoarjo General Hospital 1 month before coming to the ER and had undergone an upper gastrointestinal endoscopy or EGD. The findings showed that in the lower third of the esophagus, the mucosa was bizarre and appeared ulcerated, the lumen was narrow with a hard consistency that bled easily, and the scope could not pass through the lumen (Figure 1). The EGD results concluded that the patient had a tumor in the lower third of the esophagus leading to malignancy. A tissue biopsy was performed during the EGD and the results of histopathological examination of the esophageal tissue revealed tissue without a lining epithelium with dense inflammatory cells that were predominately neutrophils, histiocytes, plasma cells and a number of eosinophils, with many small blood vessels lined with reactive endothelium. In addition, small foci of squamous epithelial fragments with similar inflammatory cell infiltration were seen. However, no malignancy was found in the histopathological examination of the tissue preparations, and the conclusion was chronic suppurative inflammation. On this basis, the patient was then referred to the Dr. Soetomo General Academic Hospital for esophageal dilation.

At the initial physical examination, the patient was found to be moderately ill with *compos mentis* alertness. Her blood pressure was 119/84 mmHg, her pulse rate was 75 beats per minute and her respiratory rate was 18 beats per minute and she had a body temperature of 36.5 °C. The patient’s weight was 50 kg and her height was 160 cm. Her body mass index (BMI) was 19.5 kg/m^2^ and the patient gave the impression of adequate nutrition. On head examination, the conjunctiva was not anemic, the sclera was not icteric, and the pupil was isochorous. Normal tonsils were observed and no sore throat was evident. In the neck area, no dilated veins were found; the trachea was centrally located, no increase in jugular venous pressure was found. The chest appeared symmetrical, in a static and dynamic state, with normal left and right lung fremitus, no crackles or wheezing was heard. On cardiac examination, the *ictus cordis* was not visible or palpable; on percussion, the left border of the heart was at the fourth intercostal space 1 cm lateral to the left mid-clavicular line and the right border of the heart was at the fourth intercostal space of the right sternal line. The first and second heart sounds were within the normal limits, and no murmurs or galops were heard. On abdominal examination, inspection found a flat abdomen; on auscultation, bowel sounds were within the normal limits; on palpation, there was epigastric tenderness, there was no enlargement of the liver or spleen, no lump was palpable, and there was no kidney ballottement. On percussion, there was a tympanic sound throughout the abdominal field. Extremities were warm and no limb edema was observed.

Laboratory examinations in the ER revealed a hemoglobin concentration (Hb) of 11.7 g/dL, hematocrit (HCT) concentration of 35.1%, mean corpuscular volume (MCV) of 83 fL, mean corpuscular hemoglobin (MCH) of 27.7 pg, mean corpuscular hemoglobin concentration (MCHC) of 33.3 g/dL, leukocyte count of 5670/µL with neutrophils 42.1% and lymphocytes 47.4%, and platelet count of 257,000/µL. The random blood glucose (RBG) was 82 mg/dL, blood urea nitrogen (BUN) was 4.0 mg/dL, serum creatinine was 0.6 mg/dL, serum glutamic oxaloacetic transaminase (SGOT) was 57 U/L, serum glutamic pyruvic transaminase (SGPT) was 37 U/L, serum albumin was 2.88 g/dL, direct bilirubin was 0.25 mg/dL, sodium was 150 mmol/L, potassium was 2.3 mmol/L and chloride was 116 mmol/L. Examination of the hemostasis panel showed an activated partial thromboplastin time (APTT) of 24.5 s and a partial thromboplastin time (PPT) of 15.7 s. Arterial blood gas analysis with free air showed pH 7.44, pCO_2_ 43, pO_2_ 104, HCO_3_ 29.2 with a base excess (BE) of 5.0 and oxygen saturation (SO_2_) of 98%. Additionally, HbsAg, anti-hepatitis C virus (HCV), and anti-human immunodeficiency virus (HIV) rapid test results were non-reactive. A chest X-ray (CXR) performed in the ER showed traces of pulmonary inflammation without abnormalities in the heart and no visualized metastatic processes in the lungs and bones.

Based on the medical history, physical examination and supporting examinations mentioned above, the patient was diagnosed with dysphagia due to a suspected esophageal tumor with hypokalemia and hypoalbuminemia. The patient was scheduled to be hospitalized and to receive an intravenous fluid drip (IVFD) containing Triofusin500^®^:WidaKN2^®^:Kalbamin^®^ 1:1:1 with a total of 1500 mL administered every 24 h. She was also scheduled to receive an intravenous injection of 30 mg of lansoprazole every 12 h, an injection of 10 mg of metoclopramide every 8 h and 15 mL of KCl syrup every 8 h. To meet the nutritional needs of the patient, a 150 mL milk diet was also administered every 4 h (containing a total energy of 1200 kcal/day). The patient was then scheduled to have another EGD for further evaluation and confirmation of previous EGD results from Sidoarjo General Hospital, as well as a re-reading of tissue preparations in the form of paraffin blocks from Sidoarjo General Hospital.

## 3. The Disease Course and Treatment Progression

### 3.1. Treatment Day-2

The patient continued to complain of swallowing difficulty and vomiting whenever she tried to drink milk. Because there was no food or drink that could be administered orally and parenteral nutrition through peripheral veins was inadequate to support the patient’s nutritional needs, it was decided to install a central venous catheter (CVC). The CVC on the right clavicle was inserted in the ER and from then on, the patient was completely fasted and received total parenteral nutrition (TPN) in the form of Clinimix^®^:Aminofluid^®^ 1:1 in a total amount of 1500 mL every 24 h via the first port and KCl premix 50 meq in 500 mL of 0.9% NaCl every 24 h via the second port. Other intravenous drugs were continued, but the KCl syrup was discontinued due to the patient’s dysphagia.

### 3.2. Treatment Day-4

On the fourth day of treatment, the patient underwent a cardiac examination and evaluation of the cardiac risk index (CRI) to prepare for the EGD. The electrocardiographic (ECG) examination revealed a sinus rhythm of 79 beats/minute with normal frontal and horizontal axes, as well as a T-wave inversion in V1–V4. The cardiology peer assessment concluded that the heart condition was stable with good functional capacity. The patient was then classified into CRI class I with a 3.9% risk of death, myocardial infarction or cardiac arrest within 30 days. However, the T-wave inversion that appeared on the ECG could have been due to hypokalemia. On this basis, hypokalemia correctional therapy was escalated from KCl premix 50 meq in 500 mL 0.9% NaCl every 24 h to KCl premix 50 meq in 500 mL 0.9% NaCl every 8 h via CVC.

### 3.3. Treatment Day-5

The evaluation of serum electrolytes showed improvement with serum sodium 138 mmol/L, potassium 4.7 mmol/L and chloride 113 mmol/L, and the serum albumin was 2.64 g/dL. To improve the patient’s hypoalbuminemia, the TPN in the first port of the CVC was modified to Clinimix^®^:Aminofluid^®^:Kalbamin^®^ 1:1:1 per 24 h and because the hypokalemia had been corrected, the administration of KCl premix was terminated on the fifth day of treatment.

### 3.4. Treatment Day-9

**Figure 2:**
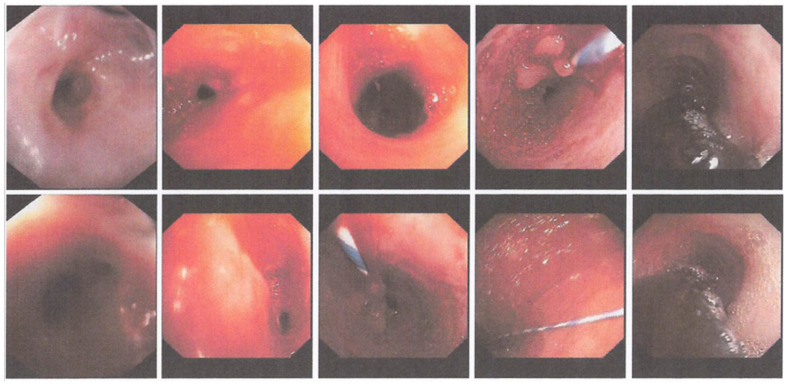
The first round of EGD performed at the Dr. Soetomo General Academic hospital showing a narrowing of esophageal lumen, which is consistent with the appearance of a corrosive stricture.

The patient underwent an EGD procedure on the ninth day of treatment. The results (Figure 2) showed that a 12 mm endoscope could enter the esophagus through the oral cavity and reach as far as 25 cm before resistance was felt. Then, the endoscope was replaced with a smaller (6 mm) scope which was able to enter the esophagus as far as the narrowing site. A narrowing of the esophageal lumen with the thickening of the mucosa was seen, and this matched the appearance of a corrosive stricture. Subsequently, the narrowing was dilated using a 9 mm Savary bougie slowly for one minute, and minimal bleeding was seen in the stricture location. Thereafter, a 6 mm scope could enter the gastric cavity. Minimal bleeding was observed. Because the endoscopic appearance matched that of a corrosive stricture, the patient was confronted by the operator and admitted that she had worked as a retail gasoline seller and often inhaled and swallowed gasoline little by little when selling. On this basis, the patient was diagnosed with esophageal stricture due to ingestion of a corrosive substance (gasoline) and was advised to undergo a second esophageal dilation a week later.

### 3.5. Treatment Day-10

After the first esophageal dilation on the ninth day of treatment, the patient felt that the barrier to swallowing in her throat was greatly reduced. The patient claimed to be able to drink water and milk without vomiting. From the physical examination, the general condition of the patient seemed adequate, with compos mentis alertness and a GCS of E4V5M6. The examination of vital signs showed a blood pressure of 114/77 mmHg, pulse rate of 85 times/min, respiratory rate of 18 times/min, body temperature of 36.5 °C and a 99% oxygen saturation with room air. A CBC on the tenth day of treatment showed Hb 9.8 g/dL, HCT 29.9%, MCV 83.1 fL, MCH 27.2 pg, MCHC 32.8 g/dL, leukocyte count 4130/µL with 55.0% neutrophils and 30.0% lymphocytes, and a platelet count of 189,000/μL. The clinical chemistry examination obtained BUN 15.9 mg/dL, serum creatinine 0.5 mg/dL, uric acid 1.8 mg/dL, SGOT 16.1 U/L, SGPT 10 U/L, serum albumin 3.19 g/dL, total bilirubin 0.40 mg/dL, direct bilirubin 0.20 mg/dL, sodium 129 mmol/L, potassium 3.6 mmol/L and chloride 104 mmol/L. The results of the patient’s hemostasis panel examination were as follows: APTT 25.7 s and PPT 13.9 s. Arterial blood gas analysis with free air showed pH 7.32, pCO_2_ 44, pO_2_ 99, HCO_3_ 22.7 with BE −3.4 and SO_2_ 97%.

The re-assessment of histopathological preparations (i.e., paraffin block) from Sidoarjo General Hospital showed pieces of tissue without lining epithelium. In the stroma, a dense layer of inflammatory cells, lymphocytes, histiocytes, plasma cells, neutrophils and eosinophils were seen. Among them, there was a proliferation of blood vessels lined with a layer of endothelium. Taken together, this picture suggested chronic inflammation.

### 3.6. Treatment Day-11

**Figure 3:**
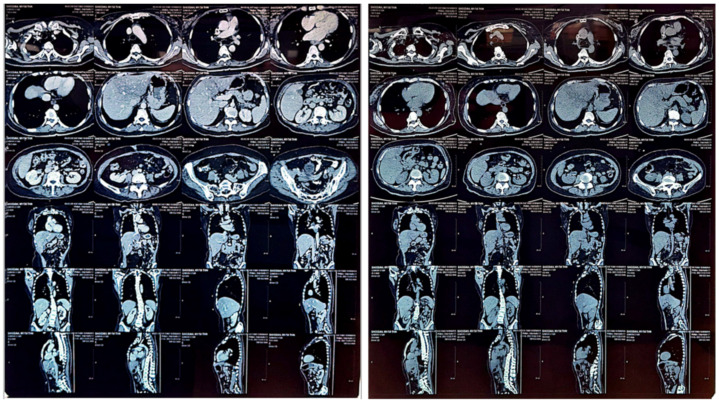
The thoracolumbar MSCT also revealed narrowing of esophageal lumen by about +/−4.5 cm.

The results of thoracolumbar axial reformatted coronal and sagittal slices on multislice computed tomography (MSCT), with and without contrast, showed diffuse, symmetrical thickening of the esophageal lumen by about +/−4.5 cm at the height of thoracic vertebrae bodies (VTh) 7 to 9, which narrowed the esophageal lumen and caused dilation of its proximal esophageal lumen. A cyst (14 HU) with dimensions of +/−1.4 × 1.6 cm in the left adrenal gland was seen. The liver was observed to be of normal size and density, with no intrahepatic bile duct (IHBD)/extrahepatic bile duct (EHBD) dilation. The portal vein/hepatica appeared normal, and no masses/nodules were seen. The GB was of normal size and density, and no masses/stones/cysts were seen. The pancreas was of normal size and parenchyma density, and no masses/cysts were seen. The spleen was of normal size and parenchyma density, and no masses/cysts were seen. The right and left kidneys were of normal size and normal parenchymal density with no ectasia of the pelvicalyceal system. Cysts (20 HU) with dimensions of +/−0.7 × 1.1 cm were seen in the lower pole of the right kidney and +/−0.9 × 0.8 cm in the lower pole of the left kidney. No extraluminal free fluid density was seen in the right and left abdominal cavity or pleural cavity. There was a lymph node in the right upper paratracheal region with a size of +/−0.8 cm. No visible osteolytic/osteoblastic process was observed. Double curve scoliosis was seen, with convexity of the thoracic vertebrae to the right and lumbar to the left. Osteophytes of the thoracic and lumbar vertebral bodies were visible. The patient appeared to have had a CVC attached with a distal tip in the right atrium. There was an appearance of fibrosis accompanied by dilation of the cylindrical type of bronchus in the apical segment of the superior lobe of the right lung. Based on these findings, it was concluded that there was diffuse, symmetrical thickening of the esophageal lumen by +/−4.5 cm as high in the body as VTh 7 to 9 which narrowed the esophageal lumen and caused dilation proximally (Figure 3). These findings indicated esophageal stricture, a left adrenal cyst, a bilateral kidney cyst, former lung inflammation, bronchiectasis, dextroscoliosis thoracalis and levoscoliosis thoracalis, as well as thoracolumbar spondylosis.

### 3.7. Treatment Day-14

The patient underwent a second EGD and esophageal dilation. The patient had no complaints and was able to drink water and milk without vomiting. From the physical examination, the general condition of the patient seemed adequate. The results of the examination of vital signs showed a blood pressure of 125/73 mmHg, pulse rate of 104 times/min, respiratory rate of 18 times/min, body temperature of 36.5 °C and a 99% oxygen saturation with room air.

Laboratory test results on the 12th day of treatment (2 days before the procedure) showed Hb 9.4 g/dL, HCT 28.4%, MCV 85.8 fL, MCH 28.4 pg, MCHC 33.1 g/dL, leukocyte count 3680/µL with 42.4% neutrophils and 39.7% lymphocytes, and platelet count 197,000/µL, and the clinical chemistry examination obtained BUN 15.3 mg/dL, serum creatinine 0.5 mg/dL, SGOT 20.8 U/L, SGPT 14 U/L, serum albumin 3.17 g/dL, sodium 130 mmol/dL. l, potassium 3.8 mmol/L and chloride 101 mmol/L.

**Figure 4:**
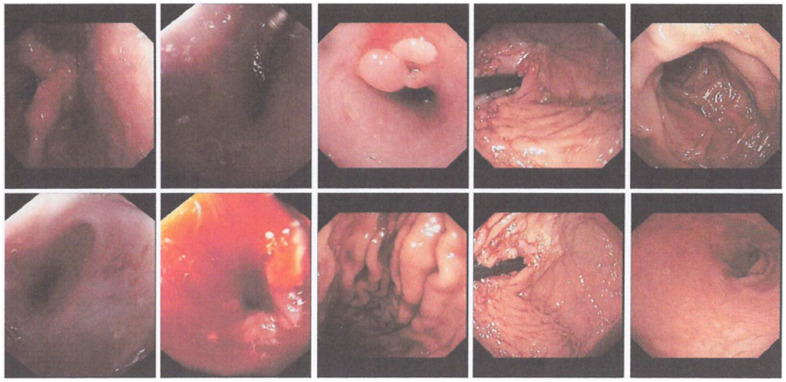
The second round of EGD performed at the Dr. Soetomo General Academic hospital.

The second EGD report (Figure 4) showed that the scope entered the oral cavity (with scope 1.2) and a narrowing was seen at the insertion of 30 cm from the incisor. Subsequently, dilation was carried out using an 11 mm Savary-Gilliard. After dilation, scope 1.2 could not enter. Scope 0.6 entered from the oral cavity, erosion and bleeding were seen in the dilated area, and a polypoid mass was seen at 35 cm insertion (near the esophagogastric junction). Then, a biopsy was performed on the mass. In the gastric mucosa, no abnormalities were seen, and no erosions/ulcers/masses were seen, whereas in the duodenum, the D1 and D2 villi were intact, and the mucosa was not visible. From the results of the EGD, it was concluded that there was a stricture of the lower third of the esophagus that had been dilated with an 11 mm Savary-Gilliard and a distal esophageal polypoid mass. The patient was scheduled for a third EGD and dilation within 2 weeks.

### 3.8. The Third EGD and Dilation (2 Weeks after the Second EGD)

After the second dilation, the patient was discharged and received further treatment at the gastroenterology outpatient clinic at the Dr. Soetomo General Academic Hospital. The patient informed that she could eat soft foods (e.g., porridge) and drink without choking or vomiting. The results of the esophageal tissue biopsy taken during the second EGD showed pieces of polypoid-shaped tissue covered with squamous epithelium, which appeared to be intact. In the stroma, lymphocytes, histiocytes, plasma cells and a few eosinophils were seen. No dysplasia was seen; no intestinal metaplasia was seen. There was no specific process or signs of malignancy. Based on these findings, it can be concluded that there was non-specific chronic esophagitis.

**Figure 5:**
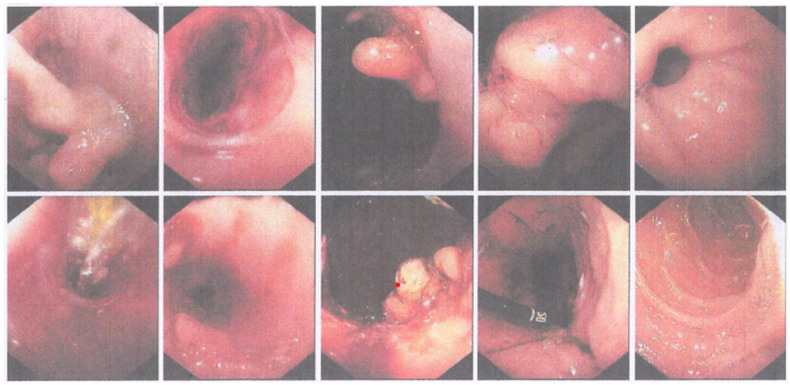
The third round of EGD performed at the Dr. Soetomo General Academic hospital.

Two weeks after the second EGD, the patient returned for the third EGD (Figure 5) and obtained the following results: the scope could go through the oral cavity up to D2. In the esophagus, there was a narrowing of the esophageal lumen starting 25–30 cm from the incisors. Dilation was carried out using 1ATM/2ATM/3ATM CRE balloons for 1 min each. Post dilation, mucosal break was visible, and no active bleeding was seen. There were two polyps in the distal esophagus. On the gastric cardia, a mass resembling granulation tissue was seen (the red dot in Figure 5), and a biopsy was performed. The fundus and body of the stomach showed no abnormalities. In the gastric antrum, an ulcer was seen with granulation tissue and cicatricial tissue, and a biopsy was performed. Normal pyloric ostium was observed. In the duodenum, the D1 and D2 villi were intact. Based on these findings, it was concluded that there was an esophageal stricture due to corrosive injury that had been dilated, as well as esophageal polyps, masses (granulation impressions) on the cardia and ulcers on the gastric antrum. Proton pump inhibitors and mucoprotectors were advised, as well as a high-protein coarse porridge diet and the patient was scheduled for another round of esophageal dilation within 2 weeks after the third EGD.

### 3.9. The Fourth EGD and Dilation (2 Weeks after the Third EGD)

After the third EGD, the patient had no further swallowing issue. The results of the histopathological examination of gastric tissue taken during the third EGD revealed pieces of gastric mucosal tissue covered with glands with shortened (eroded) gastric pits. In the lamina propria, lymphocyte inflammatory cells and 1–2 plasma cells could be seen. Locally, glandular foci were covered with epithelium with hyperchromatic nuclei. The submucosal layer was composed of fibrous connective tissue with proliferation of muscle tissue accompanied by infiltration of lymphocyte cells. There were no signs of malignancy in any tissues, which indicated the presence of granulation tissue accompanied by reactive bleeding in the gastric mucosal epithelium.

**Figure 6:**
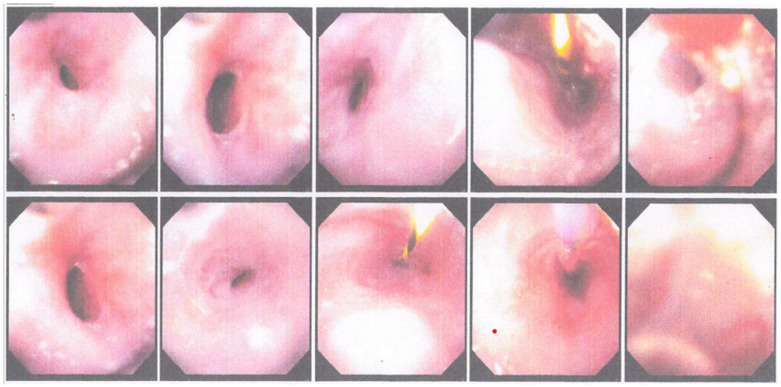
The fourth round of EGD performed at the Dr. Soetomo General Academic hospital.

During the fourth EGD session, the scope entered through the oral cavity at a 25 cm insertion. Thereafter, an esophageal stricture was detected and could not be passed by a 12 mm scope. The insertion of a 0.035 guidewire and dilation using an 8 mm balloon for 2 min were performed, followed by 12 mm balloon dilation for 2 min. Mucosal tears were visible. The lumen could not be passed by a 12 mm scope (Figure 6). In this patient, it was concluded that there was a complex esophageal stricture, which had been dilated with 8 mm and 12 mm balloons. The patient was advised to eat refined porridge and undergo another round of esophageal dilation within 1 week.

## 4. Discussion

Severe corrosive injury can induce upper gastrointestinal stricture typically three weeks post ingestion. Barium contrast swallow or contrast fluoroscopy and EGD are important initial examinations to determine further management strategies for upper gastrointestinal stricture [1,9]. Contrast fluoroscopy is only performed in patients who develop complex strictures or when endoscopy is not optimal due to excessive narrowing of the lumen, whereas EGD is more commonly recommended because it provides overall information about the anatomy of the esophagus and not only establishes the diagnosis of stricture, but also allows for mucosal biopsies and can provide an opportunity for therapeutic dilation of strictures when indicated [1]. In addition, CT scanning is also important to exclude suspected esophageal perforation due to corrosive materials that are caustic, or for strictures that are suspected to be related to esophageal malignancy (for staging purposes) [1]. This was consistent with the case presented above, where EGD was the chosen diagnostic tool employed to identify the esophageal stricture, followed by esophageal dilation as the main treatment modality.

Pathophysiologically, corrosive injury activates an inflammatory response in the affected gastrointestinal tract, followed by thrombosis of the arterioles and venules leading to ischemic necrosis. Then, fibroblasts are recruited and the reparation of the damaged mucosa begins thereafter. The stricture usually develops in the third week and the formation is complete several months after exposure. From the third week onwards, scar tissue retraction leads to stricture formation and shortening of the gastrointestinal tract. At this point, the lower esophageal sphincter pressure decreases and allows gastroesophageal reflux to occur. As a result, repeated exposure to gastric acid will accelerate the formation of strictures [9]. This was consistent with the case above because in the histopathological examination of the biopsy sample of the esophageal tissue, inflammatory cells were predominantly neutrophils, histiocytes, plasma cells and a number of eosinophils, with many small blood vessels lined with reactive endothelium, which was identical to the picture of chronic inflammation. In addition, during endoscopy, the mucosa was fragile and bled easily, reflecting the fragility of the esophageal wall exposed to corrosive materials. Clinically, the patient also had complaints of epigastric pain and vomiting every time she ate/drank, so it is likely that gastric acid reflux into the esophagus will further accelerate the development of esophageal strictures.

Management of esophageal strictures includes prevention and management of the causes of strictures, for example, in esophageal strictures caused by corrosive substances, prevention of repeated exposure to corrosive substances must be carried out. Then, dilation of the esophageal stricture can be performed to restore the patency of the narrowed esophageal lumen. Dilation using an endoscopic bougie and a balloon is the key to managing esophageal strictures [9]. The bougie-type or mechanical pusher-type dilators usually come in different sizes and are made of different materials, such as rubber. Maloney’s bougie can be passed freely without using a guidewire. Meanwhile, the Savary-Gilliard bougie has a guidewire to help it travel through the upper gastrointestinal tract. The balloon-type dilator has a way of working where the expansion of the balloon will produce a radial force that can widen the lumen [1]. Mercury-weighted rubber bougies (e.g., Maloney dilators) are commonly used for mild-to-moderate degrees of simple esophageal strictures, whereas balloon dilators (hydrostatic and pneumatic) and wire-guided polyvinyl bougies are standard modalities for more complex esophageal strictures (Table 1). A study conducted in the United States involving 348 esophageal dilation procedures over 4 years compared the performance of three methods of esophageal stricture dilation: Maloney, Savary-Gilliard and balloon (hydrostatic and pneumatic types). As a result, four incidents of esophageal perforation were reported with the use of Maloney’s bougie without fluoroscopic guidance and all of them occurred in complex esophageal strictures, whereas no incidents of perforation were reported with the use of the Savary-Gilliard dilator or balloon [10]. Of note, endoscopic-associated iatrogenic perforation is a major cause of esophageal perforation, accounting for more than half of all reported cases of esophageal perforation [11].

In this case, esophageal dilation with a Savary-Gilliard bougie was used in the first and second EGD, and a controlled radial expansion (CRE) endoscopic balloon was used in the third and fourth EGD. The patient’s stricture can be classified as a complex stricture because after four dilation sessions, patency of the esophagus has not been achieved (the lumen still cannot be passed by the 12 mm scope) and additional episodes of EGD and dilation are still needed.

A previous study mentioned the superiority of the Savary-Gilliard bougie over the Maloney, arguing that the Savary-Gilliard bougie provides greater assurance that the dilator will follow the contour of the esophageal lumen, thereby reducing the risk of perforation. In addition, wire-guided dilators offer a potential effect of radial and longitudinal dilation, depending on whether additional alternating movements are made after the initial static radial dilation. When using a Savary-Gilliard bougie, fluoroscopic assistance is recommended to monitor the guidewire position, which should be targeted at least 30 cm below the lowest point of the stricture. Usually, the distal end is positioned in the gastric antrum along the greater curvature of the stomach [12].

Through-the-scope (TTS) balloon inflation is usually performed under direct endoscope visualization, using a balloon dilator that is lowered through the endoscope channel. The center of the balloon should be centered at the narrowest point in the stricture with a dilation pressure ranging between 30 and 45 psi, varying in relation to the size of the balloon. When compared to the Savary-Gilliard, the TTS balloon dilator does not provide longitudinal compressive force because it is positioned in a static position between strictures during dilation. In addition, it is important to know the full anatomy (e.g., length and angulation) of the esophagus before balloon dilation is performed. The balloon must completely cross the stricture to avoid asymmetrical pressure across the stricture area, which can increase the risk of perforation [12]. Dilation using an endoscopic balloon is recommended in certain conditions where longitudinal pressure may be harmful, for example, in epidermolysis bullosa. In conditions where there is a tear of the esophageal mucosa (mucosal tear), it is important to carefully choose the size of the dilator, the target of the dilator and the time of dilation. It is said that dilation with an endoscopic balloon may be more advisable than using a rigid dilator in conditions with impaired continuity of the esophageal mucosa [12]. However, to date, there is no clear difference regarding the effectiveness and safety of the Savary-Gilliard bougie when compared to the endoscopic balloon (TTS) for the treatment of benign esophageal strictures [2,12]. However, the dilation of esophageal stricture due to corrosive injury using the Savary-Gilliard bougie rarely needs fluoroscopy, has shorter duration and is more economical than balloon endoscopy [13]. Ideally, the best interval between initial dilation sessions is between 2 and 4 weeks. After the goal of estimating the optimal diameter is achieved, the interval can be increased based on the patient’s expectations of the dysphagia complaints they are experiencing [12]. In the case study presented, erosion and bleeding were seen in the dilated area during the second EGD, so the dilation method was changed from the Savary-Gilliard bougie to the CRE balloon to prevent further trauma to the esophageal mucosa. Esophageal dilation in this case was performed within 1 week between the first and second dilations, followed by an interval every 2 weeks for the next episodes of dilation.

Esophageal stricture develops over time, and the prognosis depends on the timing of evaluation and treatment, and the underlying cause of the stricture. Although esophageal dilation is the first line of management in cases of benign esophageal stricture, there is a 10–40% chance of restenosis [1,14]. A stricture is considered recurrent if there is an inability to maintain a satisfactory luminal diameter for 4 weeks after achieving the target diameter of 14 mm. Whereas, a stricture is said to be refractory if the dysphagia score remains to be two (can only eat soft foods) or more as a result of the inability to achieve a diameter of 14 mm in five dilation sessions, which are carried out at intervals of every 2 weeks [1]. In the presented case, the patient was only able to eat soft foods (e.g., porridge) until the end of the fourth dilation session, so it is very likely that the patient had a refractory esophageal stricture even though she had not met the number of sessions (i.e., at least five sessions of dilation) criteria.

Stricture recurrence is a serious problem that has the potential to increase the risk and cost of treatment. The prevalence of recurrent esophageal stricture is 11.1 per 100 person-years [3]. Predictors for stricture recurrence include the presence of complex stricture, persistent epigastric pain symptoms, presence of non-peptic stricture and undiagnosed eosinophilic esophagitis. Patients with long, narrow strictures are most likely to require repeat dilation. There is no clear limit on the number of dilation sessions needed by a patient [14]. However, one study found that “bougination” or bougie dilation of esophageal stricture due to corrosive injury has a low clinical success (i.e., being able to eat a normal diet 2 months after dilation without any special procedure) rate (approximately 22.5%), when compared with other causes of strictures. The clinical success rate was significantly higher in patients with a stricture length of less than 2 cm (47.2%), those with pre-procedure dysphagia on a semi-solid or soft diet (51.3%) and those with a dilation of 13 mm or more (46.1%) [15]. In the presented case, based on the results of the MSCT scan, it was found that the narrowing of the esophagus was 4.5 cm long and dilation with serial bougie and ballooning up to the fourth session was not able to dilate the esophagus for at least 12 mm. In addition, in the pre-procedure (i.e., the first EGD), the patient could not even drink water, and the cause of the stricture was gasoline which contains corrosive substances, so it is very likely that the clinical success of repeated dilation in this patient is very small and will require repetitive dilation and other adjuvant treatments.

In general, peptic strictures have an excellent prognosis when treated promptly with endoscopic dilation and long-term PPI therapy. To improve the prognosis in terms of reducing stricture recurrence, intramural steroid injection therapy or oral steroid therapy has been used and has shown promising clinical results [1,14]. An analysis of 13 studies involving 361 subjects with corrosive esophageal injuries found that steroid therapy was not beneficial in mild corrosive injuries but could be useful in preventing strictures in moderate and severe corrosive injuries [16]. Although the mechanism of action of steroids on strictures is not completely understood, it is supposed that steroids can affect collagen deposition and increase its breakdown, thereby reducing the formation of fibrous tissue [14]. Stents are primarily used in cases of benign strictures where repeated dilation is inadequate and where symptom control is poor. In cases of malignant strictures, the prognosis depends on the type of cancer, tumor invasion, and stage of disease. Surgical resection shows a better prognosis for cancer that has not invaded the lymph nodes and surrounding tissues. In malignant strictures, stent placement can be used as palliative therapy in cases of advanced cancer or as temporary therapy in cases of ongoing neoadjuvant treatment [1].

## 5. Summary

Here, we reported the case study of a patient with complex (refractory) esophageal stricture due to chronic gasoline ingestion. The patient initially complained of difficulty swallowing which was marked by vomiting a few seconds after eating and drinking. The patient then underwent two sessions of EGD and esophageal dilation during hospitalization and was scheduled to undergo another round of EGD/dilation within 2 weeks of the last esophageal dilation session. During discharge, the patient was able to eat soft food (e.g., porridge) and drink without vomiting. During the next EGD procedure, an esophageal stricture together with esophageal polyps, masses (i.e., granulation tissue) on the cardia and ulcers on the gastric antrum were observed. Then the patient underwent another esophageal dilation, this time with a CRE balloon, and a histopathological examination of gastric tissue was performed. To date, the patient has undergone at least four EGD sessions with repeated esophageal dilation. Vomiting when eating/drinking and the swallowing issue have significantly been reduced, even though endoscopically the lumen of the esophagus is still narrow (the scope was unable to be traversed by a 12 mm scope).

## Figures and Tables

**Table 1:** Differences between simple and complex esophageal strictures.

	Simple Stricture	Complex Stricture
Endoscopy scope access	Yes	No (usually)
Size	Short (<2 cm)	Long (>2 cm)
Focal	Yes	No
Angulation/irregularity	No	Yes
Cause	Peptic, Shatzki’s ring, anastomosis, pill-induced	Caustic ingestion, malignancy, photodynamic therapy, radiation
Recommended dilation method	Balloon or rigid dilator	Rigid dilator
Fluoroscopy	Rarely needed	Recommended
Frequency of dilation	1–3 (commonly)	≥3
Recurrence risk	Low	High

## Data Availability

Data is unavailable due to privacy of the patient.
